# Preliminary investigation of kinetin, polyethylene glycol, iron sulphate, and water priming effects on the phytochemical accumulation and antimicrobial activity of *Corchorus trilocularis* L

**DOI:** 10.3389/fpls.2026.1845397

**Published:** 2026-06-15

**Authors:** Frank Phasha, Sweetvoice Mzinyane, Nkateko Phasha, Phumzile Mkhize

**Affiliations:** Department of Biochemistry, Microbiology and Biotechnology, University of Limpopo, Mankweng, South Africa

**Keywords:** antibacterial activity, antioxidant activity, *Corchorus trilocularis*, phytochemical accumulation, seed priming

## Abstract

**Introduction:**

This study preliminarily investigated how seed priming with kinetin (K), polyethylene glycol (PEG), iron sulphate (FeSO_4_), and water (W) can modulate phytochemical accumulation, antioxidant capacity, and antibacterial activity of *Corchorus trilocularis* across vegetative and flowering growth stages. *C. trilocularis* was selected due to its reported nutritional and medicinal importance.

**Methods:**

Methanolic leaf extracts were analysed using standard phytochemical, antioxidant, and antibacterial assays.

**Results:**

Multivariate analysis revealed that both priming treatment and growth stage significantly influenced phytochemical accumulation (p< 0.001). Phytochemical levels were generally higher at the flowering stage. FeSO_4_ priming consistently produced the greatest phytochemical accumulation, increasing flavonoids (70%), phenolics (42-50%), and tannins (~40%) relative to the control at flowering. This enhancement may be attributed to priming-induced stress signalling, which stimulates secondary metabolite biosynthesis. FeSO_4_ and PEG treatments showed 20-30% greater radical scavenging activity. Significant positive correlations were observed between priming treatments and phytochemical accumulation. A treatment-dependent antibacterial activity against Staphylococcus aureus was observed, with PEG-primed plants showing the strongest activity (MIC 0.14 mg/mL, MBC 1.18 mg/mL), followed by FeSO_4_ (MIC 0.16 mg/mL, MBC 1.25 mg/mL), whereas no activity was observed against *Pseudomonas aeruginosa*.

**Discussion:**

The lack of activity against *P. aeruginosa* may be associated with its Gram-negative cell wall structure and intrinsic resistance mechanisms. No antibacterial activity was observed at the vegetative stage. Overall, extracts from the flowering stage exhibited greater bioactivity than those from the vegetative stage. These findings demonstrate that seed priming and plant developmental stage are key determinants of phytochemical accumulation and bioactivity. This highlights seed priming as a strategy to enhance the pharmacological potential through optimised cultivation and harvest management.

## 1 Introduction

*C. trilocularis* L. is an underutilised leafy vegetable widely distributed in tropical and subtropical regions, where it is primarily cultivated under low-input, rain-fed conditions for both nutritional and medicinal purposes. The species is recognised for its high nutritional value and diverse phytochemical composition, including flavonoids, phenolics, tannins, and alkaloids, which contribute to its medicinal properties (The species is recognised for its high nutritional value and diverse phytochemical composition, including flavonoids, phenolics, tannins, and alkaloids, which contribute to its medicinal properties (Kumari et al., 2019; Hossen et al., 2022; Kudumela et al., 2024; Dube et al., 2019). However, this variability and generally suboptimal accumulation of phytochemicals limit the consistent medicinal and nutritional value of the species, highlighting the need for strategies to enhance its bioactive potential (Abdel-Razek et al., 2022; Ncube et al., 2012).

(Maina et al., 2011; Hossen et al., 2022). The increasing prevalence of antibiotic-resistant pathogens, such as *Staphylococcus aureus* and *Pseudomonas aeruginosa*, underscores the need for alternative, plant-based antimicrobial strategies (El-Sayed et al., 2025; Hu and Chua, 2025). Traditionally, *Corchorus* species have been used to manage ailments such as infections, inflammation, gastrointestinal disorders, and fever, further supporting their ethnomedicinal relevance (Maina et al., 2011; Hossen et al., 2022).

However, despite these benefits, the therapeutic efficacy of *C. trilocularis* is often limited by variability and generally low concentrations of key bioactive compounds (Abdel-Razek et al., 2022). Compared to other medicinal plants, fluctuations in phytochemical accumulation due to growth stage, environmental stress, and genetic factors can reduce its effectiveness against pathogenic microorganisms (Ncube et al., 2012). This variation is partly attributed to metabolic shifts during plant development, where secondary metabolite production typically increases during the reproductive (flowering) stage to support defence and reproductive success (Li et al., 2020; Mohammadi-Cheraghabadi et al., 2021). This is particularly important in the context of infections caused by *Staphylococcus aureus* and *Pseudomonas aeruginosa*, which are major contributors to antimicrobial resistance and pose significant public health challenges. Enhancing phytochemical content is therefore essential to improve the plant’s antibacterial potency and reliability as a medicinal resource.

The use of traditional medicinal plants also faces broader challenges, including inconsistent bioactivity, overharvesting, and lack of standardised cultivation practices (Mofokeng et al., 2022; Lulesa et al., 2025; Zamani et al., 2025). These limitations necessitate the development of sustainable strategies to improve both yield and phytochemical quality. While approaches such as genetic modification, *in vitro* culture, and elicitor application have been explored to enhance phytochemical production, these methods are often costly and technically demanding, making them less accessible to smallholder farming systems (Hasnain et al., 2022; Martínez-Chávez et al., 2024; Al Nooh et al., 2025). Among these, seed priming has emerged as a simple and cost-effective approach to enhance plant performance and secondary metabolite production (Farooq et al., 2019; Paparella et al., 2015).

Priming treatments can modulate key physiological and biochemical pathways, leading to improved germination, stress tolerance, and metabolic activity (Paparella et al., 2015, and Nawaz et al., 2013). Different priming agents exert distinct biochemical effects. For example, kinetin, a cytokinin, promotes cell division and metabolic activation, potentially enhancing secondary metabolite biosynthesis (Jameson, 2023). Polyethylene glycol (PEG) induces controlled osmotic stress, stimulating antioxidant defence systems and accumulation of protective compounds (Ma et al., 2024). For example, seed priming with 30% PEG improved α-amylase activities and total soluble sugar contents under nano-ZnO stress. In addition, glutathione reductase activity, reactive oxygen species accumulation, and proline contents decreased after the PEG (Saleem et al., 2022).priming treatment in *Oryza sativa* cultivars under different nano-ZnO concentrations (Sheteiwy et al., 2016). Iron sulphate plays a critical role in enzymatic processes and redox reactions facilitate chlorophyll synthesis and phenolic metabolism (Du et al., 2023). For example, FeSO_4_ application reduces oxidative stress, including malondialdehyde content and hydrogen peroxide, by enhancing antioxidant enzymatic activity such as catalase, ascorbate peroxidase, superoxide dismutase, peroxidase, and glutathione peroxidase, as well as radical scavenging activity measured by 2,2-diphenyl-1-picryl-hydrazyl-hydrate (Saleem et al., 2022). It also increases non-enzymatic antioxidant compounds, including proline, amino acids, total soluble protein, phenolics, flavonoids, reducing and non-reducing sugars, and carbohydrates, in all cultivars of *Oryza sativa* (Saleem et al., 2022). Hydropriming (water) serves as a baseline treatment, improving seed hydration and metabolic readiness without introducing additional chemical stimuli (Velempini et al., 2003). Therefore, seed priming may influence plant chemodiversity by altering the accumulation patterns of multiple secondary metabolite classes across developmental stages, thereby potentially modifying biological activity.

Despite these advances, there is limited understanding of how seed priming strategies influence the relationship between phytochemical accumulation and antibacterial activity in *C. trilocularis*, particularly across different developmental stages. Addressing this gap is important for improving the consistency and efficacy of plant-derived antimicrobial compounds. Given these mechanisms, integrating seed priming with targeted cultivation strategies offers a promising pathway to enhance the phytochemical profile and bioactivity of *C. trilocularis*. It was hypothesised that seed priming with kinetin, polyethylene glycol, and FeSO_4_ would differentially enhance phytochemical accumulation and, consequently, improve antioxidant and antibacterial activity, particularly at the flowering stage. This study therefore evaluates the effects of different seed priming treatments (kinetin, polyethylene glycol, FeSO_4_, and hydropriming) on phytochemical accumulation, antioxidant capacity, and antibacterial activity against *Staphylococcus aureus* and *Pseudomonas aeruginosa* across distinct developmental stages. By integrating seed priming, plant developmental stage, and bioactivity assessment, this study aims to provide a practical framework for enhancing the medicinal potential of *C. trilocularis*, with implications for sustainable cultivation and utilisation of underutilised crops.

## 2 Materials and methods

### 2.1 Seed priming

Dry fruit pods of *C. trilocularis* were collected from plants growing in a home garden in the Molapi area of the Greater Mankweng district, Limpopo Province, South Africa, where the species is commonly cultivated for local use Seeds were manually separated from the pods and used for the seed priming experiments. No formal accession number or genetic characterization of the plant material was available for this study. Scarified *C. trilocularis* seeds were subjected to different priming treatments by soaking them in the following solutions: (1) distilled water, (2) 1% (FeSO_4_), (3) 3% polyethylene glycol (PEG), and (4) 5 mg/L kinetin. Seeds were immersed in the respective priming solutions for 3 hours at room temperature, while non-primed seeds served as the control. The selected priming concentrations and duration were based on preliminary optimisation trials and previously published seed priming studies reporting physiological responses within comparable concentration ranges. After priming, the seeds were rinsed five times with distilled water to remove any residual priming solution.

### 2.2 Experimental design and sample collection

Primed seeds (14 per treatment, including the control) were sown in seedling trays with removable plugs filled with moist vermiculite and maintained in a controlled growth room under a 16:8 h light: dark photoperiod at 30 °C. Vermiculite served as a low-nutrient inert growth substrate. The vermiculite was kept consistently moist until germination, and seedlings were irrigated twice weekly with tap water. Nutrient supplementation was initiated in the third week using one-quarter-strength Hoagland (KNO_3_, 1.5 mM; Ca(NO_3_)_2_·4H_2_O, 1.25 mM; MgSO_4_·7H_2_O, 0.5 mM; KH_2_PO_4_, 0.25 mM; H_3_BO_3_, 11.6 µM; MnCl_2_·4H_2_O, 2.3 µM; ZnSO_4_·7H_2_O, 0.19 µM; CuSO_4_·5H_2_O, 0.08 µM; Na_2_MoO_4_·2H_2_O, 0.03 µM; Fe-EDTA, 25 µM) solution applied as a soil drench, followed by half-strength solution (KNO_3_, 3.0 mM; Ca(NO_3_)_2_·4H_2_O, 2.5 mM; MgSO_4_·7H_2_O, 1.0 mM; KH_2_PO_4_, 0.5 mM; H_3_BO_3_, 23.0 µM; MnCl_2_·4H_2_O, 4.6 µM; ZnSO_4_·7H_2_O, 0.38 µM; CuSO_4_·5H_2_O, 0.16 µM; Na_2_MoO_4_·2H_2_O, 0.06 µM; Fe-EDTA, 50 µM) in the fourth week and full-strength solution (KNO_3_, 6.0 mM; Ca(NO_3_)_2_·4H_2_O, 5.0 mM; MgSO_4_·7H_2_O, 2.0 mM; KH_2_PO_4_, 1.0 mM; H_3_BO_3_, 46.0 µM; MnCl_2_·4H_2_O, 9.2 µM; ZnSO_4_·7H_2_O, 0.76 µM; CuSO_4_·5H_2_O, 0.32 µM; Na_2_MoO_4_·2H_2_O, 0.12 µM; Fe-EDTA, 100 µM) from the fifth week onwards. Nutrient concentration was gradually increased to prevent osmotic stress in young seedlings and to align nutrient availability with developmental demand, allowing progressive acclimation of the root system and supporting optimal vegetative growth. Six-week-old seedlings were transplanted into plastic pots containing a potting mixture composed of potting soil, coco peat, vermiculite, and sand in a ratio of 5:3:2:1 (v/v). The plants were then transferred to a greenhouse under ambient, uncontrolled environmental conditions. Although environmental variables were not fully controlled after transplantation, all treatments were maintained under the same greenhouse conditions to minimise systematic environmental bias. Regular watering and weekly nutrient application were maintained until the plants reached three months of age.

At three months, three plants per treatment were randomly selected for sampling. Sampling was conducted at the mid-point of the vegetative developmental stages to minimise developmental heterogeneity among plants and to provide representative phytochemical profiles for the growth phase. Five leaves per plant were collected beginning from the ninth node toward the apical region. This standardized sampling position was selected to minimise variation associated with leaf developmental stage and physiological age, ensuring that leaves of comparable maturity and metabolic activity were analysed. These samples were treated as biological replicates. The plants were allowed to grow until the flowering stage, after which leaf sampling was again conducted at mid-point of the flowering growth phase from the same plants used for vegetative stage sampling. The leaves were also collected from the ninth node toward the apex to maintain consistency in developmental stage across sampling periods. These were used for analysis at flowering stage.

### 2.3 Extraction of plant material

Extraction was performed to quantify on the leaves of *C. trilocularis* using methanol. Extraction was conducted on leaves collected at both the vegetative and flowering growth stages. For extraction, 50 g of milled leaf material from each treatment and the control were macerated separately in 500 mL of methanol (HPLC-grade methanol obtained from Sigma-Aldrich (South Africa)) at a 1:10 (w/v) ratio. The mixtures were agitated continuously for 24 h at room temperature using a shaking incubator machine (New Brunswick Scientific Co., Inc., Edison, NJ, USA) set at 200 rpm. The extracts were then filtered and collected in pre-weighed, labelled containers. To ensure complete recovery the extraction procedure was repeated for additional extraction cycles of three hours and one hour, respectively, and the resulting extracts were pooled. The combined extracts were subsequently concentrated by evaporating the solvent under a stream of cold air using a commercial air conditioner set at 16 °C. Following extraction and solvent evaporation, the crude extracts were transferred into sterile amber glass containers and stored at 4 °C until further analysis to minimize light- and temperature-induced degradation. The extracts were used for phytochemical and biological assays within one week of preparation.

### 2.4 Quantification of polyphenolics

#### 2.4.1 Total phenolic content

Total phenolic content (TPC) was quantified using the Folin-Ciocalteu assay following the method of Tambe and Bhambar (2014), with minor modifications. Briefly, 100 μL of each extract (10 mg/mL) was diluted with 900 μL of distilled water and combined with 250 μL of Folin-Ciocalteu reagent. After allowing the reaction to proceed for 5 min, 1.25 mL of 7% sodium carbonate solution was added, and the mixture was incubated at room temperature in the dark for 30 min. Absorbance was recorded at 725 nm using a UV-VIS spectrophotometer. Quantification was performed using a gallic acid standard curve (0.08-1.25 mg/mL), and results were expressed as milligrams of gallic acid equivalents per gram of extract (mg GAE/g). TPC values were calculated from the gallic acid calibration curve. All analyses were conducted with three biological replicates and three technical replicates.

#### 2.4.2 Total tannin content

Total tannin content (TTC) was quantified using the Folin-Ciocalteu method described by Tambe and Bhambar (2014). Briefly, 50 μL of each extract was adjusted to a final concentration of 10 mg/mL with distilled water. The solution was then mixed with 3.8 mL of distilled water and 0.25 mL of Folin-Ciocalteu reagent, followed by the addition of 0.5 mL of 35% sodium carbonate solution. The reaction mixture was further diluted to a final volume of 10 mL with distilled water, vortexed thoroughly, and incubated in the dark at room temperature for 30 min. Absorbance was measured at 725 nm using a UV-VIS spectrophotometer. Gallic acid was used as the calibration standard (0.0625–1 mg/mL), and results were expressed as milligrams of gallic acid equivalents per gram of extract (mg GAE/g). All measurements were performed using three biological replicates and three technical replicates.

#### 2.4.3 Total flavonoid content

Total flavonoid content (TFC) was determined using the aluminium chloride colorimetric method as described by Beseni et al. (2019). Briefly, 100 μL of each extract (10 mg/mL) was mixed with 100 μL of 10% aluminium chloride, 100 μL of 1 M potassium acetate, and 2.8 mL of distilled water. The reaction mixture was incubated at 25 °C for 30 min. Absorbance was measured at 415 nm using a microplate reader. Quercetin was used to construct the standard calibration curve, and results were expressed as milligrams of quercetin equivalents per gram of extract (mg QE/g). All measurements were performed in three biological replicates and three technical replicates.

### 2.5 DPPH free radical scavenging assay

The free radical scavenging activity of extracts from different treatments was evaluated using the 2,2-diphenyl-1-picrylhydrazyl (DPPH) assay, following the method described by Bondet et al. (1997) with slight modifications. Stock solutions of each extract were prepared in HPLC-grade methanol (Sigma-Aldrich, South Africa) at an initial concentration of 1250 µg/mL. Serial two-fold dilutions were then prepared to obtain concentrations of 625.00, 312.50, 156.25, 78.13, 39.06, 19.53, 9.76, and 4.88 µg/mL, each adjusted to a final volume of 1 mL. All concentrations were prepared using three biological replicates and three technical replicates. L-ascorbic acid was used as a positive control and prepared over the same concentration range as the extract standards. To each 1 mL extract or standard solution, 2 mL of 0.2 mmol/L DPPH solution (prepared in methanol) was added, and the mixtures were vortexed thoroughly. A negative control was prepared by adding 2 mL of 0.2 mmol/L DPPH solution to 1 mL of distilled water. All reaction mixtures were incubated for 30 min. During the 30 min incubation period, all reaction mixtures were incubated in the dark at room temperature to minimize light-induced degradation of the DPPH radical. Following incubation, absorbance was measured at 517 nm using a DU^®^ 730 Life Science UV/Vis spectrophotometer. The percentage radical scavenging activity (% inhibition) was calculated using the equation described by Ahmed et al. (2012).


$$\%\;Inhibition=\left[\frac{Ac-As}{Ac}\right]\times 100$$

Where,

Ac, Absorbance of control (negative) solution.

As, Absorbance of cucurbitacin solution.

### 2.6 Antimycobacterial activity

#### 2.6.1 Microorganisms used in the study

The antibacterial activity assays were conducted using two American Type Culture Collection (ATCC) reference strains, namely *Staphylococcus aureus* subsp. aureus ATCC 29213 and *Pseudomonas aeruginosa* ATCC 27853. *S. aureus* ATCC 29213 is a methicillin-susceptible quality-control strain commonly used for antimicrobial susceptibility testing, while *P. aeruginosa* ATCC 27853 is a standard quality-control strain originally isolated from a blood culture and widely used in antimicrobial susceptibility assays.

#### 2.6.2 Determination of the minimum inhibitory concentration and minimum bactericidal concentration

The method used for the detection of the extract’s minimum inhibitory concentration (MIC) was adopted from Senhaji et al. (2020) and Eloff (1998) with modification. In three biological and technical triplicates, leaf extracts from the different treatments from both the flowering and vegetative growth were prepared to an initial concentration of 10 mg/mL. These were added into the different wells of the sterile 96 NUC-ELISA plates. To these ELISA wells nutrient broth was added and successive dilutions were then carried out resulting to a to a final volume of 100 µl and concentration of 1250.00, 625.00, 312.50, 156.50, 78.13, 39.10, 19.53 and 9.76  μg/mL. Thereafter, each well was then inoculated with 100 µl of the different bacterial cultures i.e. *Staphylococcus aureus* and *Pseudomonas aeruginosa.* The plates were then covered and incubated at 37 °C for 24 hours. Ampicillin was used as the standard antibiotic. Nutrient broth was used as the negative control. After the incubation period 40 μL of 0.2 mg/mL of p-iodonitrotetrazolium chloride dissolved in sterile distilled water was added to each well and further incubated for 30 min. The presence of bacterial growth was monitored by the appearance of a violet to purple colour. The wells that remained clear were an indication of a successful inhibition of bacterial growth.

Minimum bactericidal concentration (MBC) was conducted according to Senhaji et al. (2020) with some modification. In this method the plates initially used for the MIC determination were incubated for a further 24 hours at 37 °C. Thereafter, bacterial growth was monitored and clear wells indicated inhibition. After, a 10 *μ*L of the samples were sub-cultured on nutrient agar and incubated for 24 h at 37 °C. No bacterial growth on the nutrient agar plates were an indication of a successful bactericidal. Total activity (TA) was calculated by dividing the quantity of extract obtained from 1 g of plant material by the MIC value, as previously described in antimicrobial screening studies of medicinal plants (Eloff, 1998). Total activity provides an estimate of the volume to which the extract derived from 1 g of plant material can be diluted while still retaining antimicrobial activity, thereby integrating extraction yield and antimicrobial potency into a single comparative parameter.

### 2.7 Evaluation of bacterial growth kinetics during treatment

In this study, the growth kinetics of *S. aureus* were monitored over a 24 h period in the presence of plant extracts and ampicillin as a positive control, following the method described by Jiang et al. (2011) with slight modifications. The bacterium was inoculated into nutrient broth and incubated at 37 °C for 24 h. Overnight cultures were then transferred into 20 mL of fresh nutrient broth supplemented with the test extracts or ampicillin at their different minimal inhibitory concentration. The initial optical density (OD_600_) was adjusted to 0.02 (≈1 × 10^6^ CFU/mL). Cultures were incubated at 37 °C with continuous shaking, and bacterial growth was monitored by measuring OD_600_ at 0, 1, 2, 3, 4, 5, 6, 9, 12, 18, and 24 h using a UV-Vis spectrophotometer. An untreated culture served as the negative control, while uninoculated media was used as the blank.

### 2.8 Combinational effects of plant extracts

The combined effects of plant extracts obtained from different seed priming treatments were evaluated to determine their interactions using the method described by van Vuuren and Viljoen (2011) through the calculation of the fractional inhibitory concentration (FIC). The broth microdilution assay was performed as previously described in Section 2.7.1. The FIC value for each extract combination was calculated using Equation 1:

(1) $${\text{FIC}}_{\text{A}}=\frac{MIC\;of\;A\;in\;combination}{2MIC\;of\;A\;alone},{\text{ FIC}}_{\text{B}}=\frac{MIC\;of\;B\;in\;combination}{MIC\;of\;B\;alone}$$

The sum of FICs (ΣFIC) was obtained using Equation 2:

(2) ∑FIC=FICA+FICB

The interaction was interpreted as synergistic (ΣFIC ≤ 0.5), additive (ΣFIC > 0.5-1.0), indifferent (ΣFIC > 1.0-4.0), or antagonistic (ΣFIC > 4.0).

### 2.9 Statistical analysis

All statistical analyses were performed using R statistical software (version 4.4.0 - 2024-04–24 ucrt). Descriptive statistics, including means and standard deviations, were calculated for all measured variables. Data were analysed using appropriate parametric tests, and statistical significance was determined at the 5% probability level (*p* < 0.05), unless otherwise stated. Where relevant, *post-hoc* comparisons were conducted to separate treatment means following significant effects. Graphical visualisations were also generated in R to illustrate treatment effects and variable relationships. Homogeneity of variances was assessed using Levene’s test, while equality of covariance matrices was evaluated using Box’s M test prior to MANOVA.

Pearson correlation coefficients were calculated using R statistical software to assess the strength and direction of associations among priming solutions. Statistical significance of the correlation coefficients was tested using Student’s *t*-test.

## 3 Results

### 3.1 Multivariate analysis of variance of seed priming and growth stage effects on phytochemical accumulation

A multivariate analysis of variance was conducted to assess the effects of the seed priming solution, growth stage and their interaction on the accumulation of different phytochemical accumulation. Wilks’ Lambda was used to evaluate the significance of the multivariate effects (**Table 1**). The analysis revealed a significant effect of priming solution type (Wilks’ Λ, 0.177, F(10, 88), 12.10, p< 0.001), indicating that different solutions resulted in statistically distinct multivariate responses. The multivariate effects observed for priming treatment, growth stage, and their interaction were associated with comparatively large effect magnitudes, as reflected by the low Wilks’ Lambda values obtained for each factor. Growth stage showed the strongest multivariate effect (Wilks’ Λ, 0.104), followed by the treatment × growth stage interaction (Wilks’ Λ, 0.127) and priming treatment (Wilks’ Λ, 0.177), indicating substantial contributions of these factors to variation in phytochemical accumulation. Prior to interpretation of the multivariate model, homogeneity of variance was assessed using Levene’s test. Significant heterogeneity was observed for total phenolic content (TPC; p, 0.0019) and total flavonoid content (TFC; p, 0.0003), whereas total tannin content (TTC) did not significantly deviate from homogeneity assumptions (p, 0.063). However, the balanced experimental design supported the suitability of the MANOVA framework despite moderate variance heterogeneity. Follow-up univariate analyses indicated that the significant treatment × growth stage interaction observed in the MANOVA differed among phytochemical classes. Flavonoid- and phenolic-associated responses showed comparatively stronger developmental responsiveness across priming treatments, whereas tannin accumulation exhibited comparatively lower treatment differentiation across growth stages.

**Table 1:** Multivariate analysis of variance of the effects of seed priming treatments, growth stage, and their interaction on phytochemical accumulation.

Effects	Wilks’ Λ	F	df1	df2	Significance
Phytochemicals (TT)	0.18	12.10	10	88	< 0.001**
Growth stage (TM)	0.10	75.56	5	44	< 0.001**
TT × TM	0.13	15.92	10	88	< 0.001**

Wilks’ Λ, Wilks’ Lambda statistic; df1, numerator degrees of freedom; df2 , denominator degrees of freedom; Significance codes: ** p< 0.001.

### 3.2 Effect of seed priming with kinetin, polyethylene glycol, FeSO_4_, and water on the total flavonoid, phenolic and tannin content in *C. trilocularis* at vegetative and flowering stages

**Figure 1:**
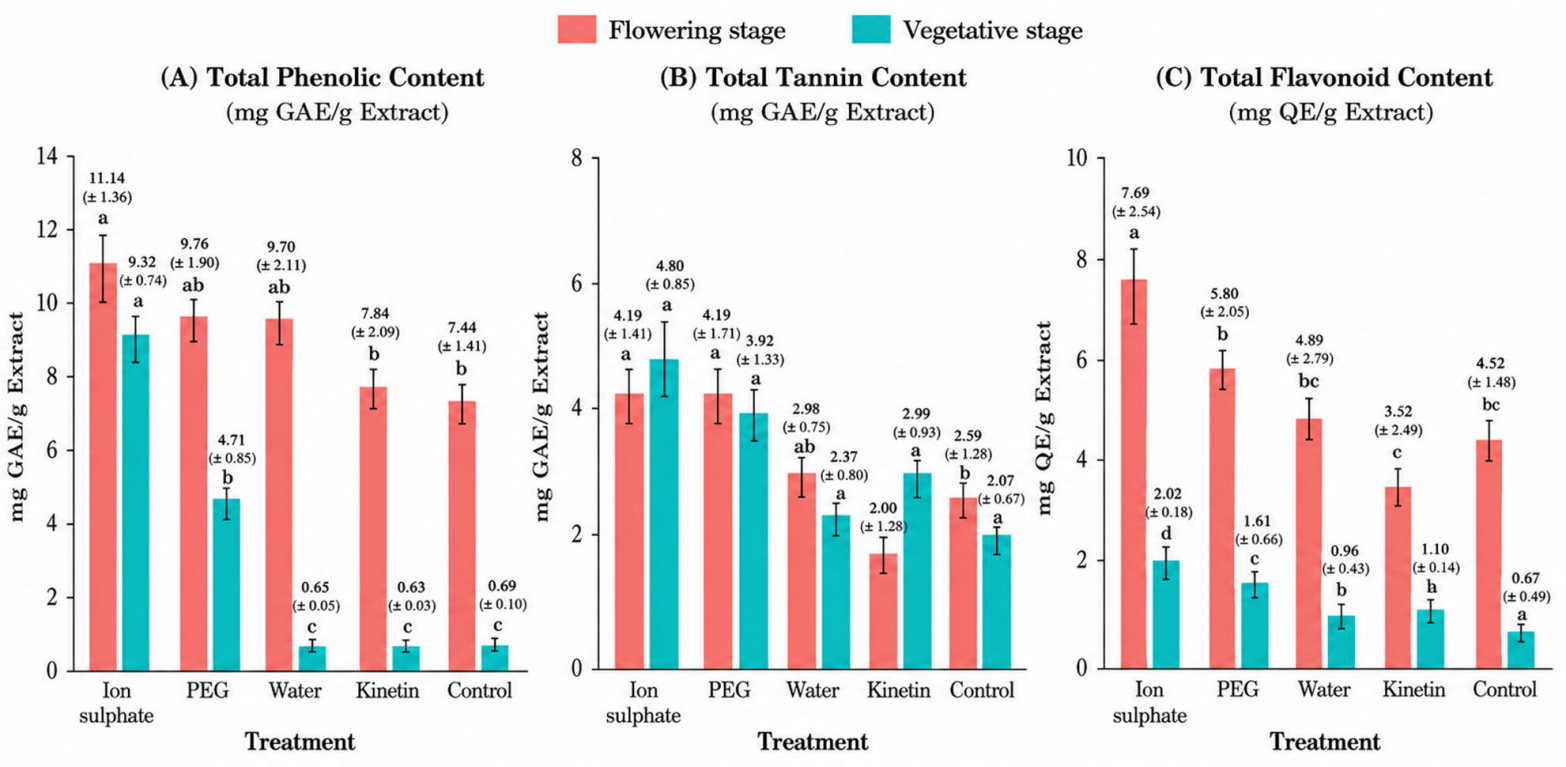
Effects of seed priming treatments on phytochemical accumulation during the vegetative and flowering growth stages of *Corchorus trilocularis*. **(A)** Total phenolic content (TPC), **(B)** total tannin content (TTC), and **(C)** total flavonoid content (TFC) measured on a dry weight basis. PEG = polyethylene glycol. Values represent mean ± standard deviation (n = 3 biological replicates). Different letters indicate statistically significant differences among treatments within each growth stage (p < 0.05), determined using one-way ANOVA followed by Tukey’s honestly significant difference (HSD) post-hoc test. Bars sharing the same letters are not significantly different (p > 0.05).

Total flavonoid content varied significantly across treatments and growth stages as indicated by the means, standard deviation and levels of significance represented by letters (**Figure 1**), During the flowering stage, the FeSO_4_ priming produced the highest flavonoid content (7.69 ± 2.53), which was significantly higher (a) than both kinetin (K; 3.52 ± 2.49) and the control (C; 4.52 ± 1.48). The control and kinetin did not differ from each other (b). Polyethylene glycol (5.81 ± 2.05) and water (4.89 ± 2.79) produced intermediate responses (ab) and were not significantly different from either the highest or lowest groups.

Total phenolic content showed strong priming solution-dependent and growth stage-dependent variation (**Figure 1**). Flowering plants generally accumulating substantially higher phenolic content than vegetative plants, except under FeSO_4_ treatment where phenolics remained high in both stages. At the flowering stage, FeSO_4_ produced the highest phenolic content (11.14 ± 1.36), significantly exceeding kinetin (7.84 ± 2.09) and the control (7.44 ± 1.41). Priming with kinetin and control formed a lower but statistically similar group (b). PEG (9.76 ± 1.90) and water (9.70 ± 2.11) showed intermediate accumulation (ab), not significantly different from either the highest or lowest treatments. In contrast, during the vegetative stage, FeSO_4_ maintained very high phenolic levels (9.33 ± 0.74; a), clearly exceeding all other treatments. PEG showed moderate accumulation (4.71 ± 0.85; b). Kinetin (0.63 ± 0.03), water (0.65 ± 0.05), and the control (0.70 ± 0.11) produced very low phenolic levels and did not differ significantly (c). These results indicate that FeSO_4_ strongly enhances phenolic accumulation irrespective of growth stage, while most other treatments show pronounced stage dependency.

Total tannin content showed less pronounced priming solution and stage differentiation compared with flavonoids and phenolics. During the flowering stage, FeSO_4_ (4.19 ± 1.41) and PEG (4.19 ± 1.71) produced the highest tannin concentrations and were statistically similar (a). Kinetin resulted in significantly lower tannin accumulation (2.00 ± 1.28; b), while the control (2.98 ± 0.96) and water (2.58 ± 0.75) showed intermediate levels (ab), not significantly different from either extreme. During the vegetative stage, no significant differences among treatments were detected, as all treatments shared the same statistical grouping (a). Although FeSO_4_ showed the numerically highest value (4.80 ± 3.66), followed by PEG (3.92 ± 2.33) and kinetin (2.99 ± 0.95), variability was high and treatment effects were not statistically distinguishable. These findings suggest that tannin accumulation is less responsive to priming and developmental stages than flavonoids and phenolics.

*Post-hoc* pairwise comparisons were further evaluated using false discovery rate-adjusted multiple comparison procedures to control for Type I error across repeated pairwise tests. The adjusted comparisons confirmed significant phytochemical differences among priming treatments and growth stages, particularly for flowering-stage flavonoid and phenolic accumulation (**Table 2**). False discovery rate -adjusted *post-hoc* comparisons showed that FeSO_4_ differed significantly from the lower accumulating treatments, while PEG, water, kinetin, and control showed partial overlap among statistical groupings (**Table 2**). During the vegetative stage, flavonoid concentrations were markedly reduced across all treatments. Ion sulphate again produced the highest flavonoid level (2.02 ± 0.18; a), followed by PEG (1.61 ± 0.66; ab). Kinetin showed moderate accumulation (1.10 ± 0.14; c), whereas the control (0.67 ± 0.50) and water (0.96 ± 0.43) produced the lowest levels. Overall, flowering plants exhibited consistently greater flavonoid accumulation than vegetative plants across all treatments.

**Table 2:** Total phenolic, total tannin, and total flavonoid contents of primed *C. trilocularis* plant extracts at vegetative and flowering stages, based on false discovery rate-adjusted *post-hoc* comparisons.

Priming solution	TPC (mg GAE/g extract)	TTC (mg GAE/g extract)	TFC (mg QE/g extract)	TPC (mg GAE/g extract)	TTC (mg GAE/g extract)	TFC (mg QE/g extract)
	Vegetative stage			Flowering stage		
FeSO_4_	9.33 ± 0.74^c^	4.80 ± 0.85^b^	2.02 ± 0.18^a^	11.14 ± 1.36^d^	4.19 ± 1.41^b^	7.69 ± 2.54^c^
Polyethylene glycol	4.71 ± 0.85^bc^	3.92 ± 1.33^b^	1.61 ± 0.66^a^	9.76 ± 1.90^d^	4.19 ± 1.71^b^	5.81 ± 2.05^c^
Water	0.65 ± 0.05^a^	2.37 ± 0.80^bc^	0.96 ± 0.34^ab^	9.70 ± 2.11^e^	2.58 ± 1.06^c^	4.89 ± 2.79^d^
Kinetin	0.63 ± 0.03^a^	2.99 ± 0.93^bc^	1.10 ± 0.43^a^	7.84 ± 2.09^d^	2.00 ± 1.28^ab^	3.52 ± 2.49^c^
Control	0.70 ± 0.10^a^	2.07 ± 0.67^b^	0.67 ± 0.22^a^	7.44 ± 1.41^e^	2.98 ± 1.33^c^	4.52 ± 1.48^d^

Values represent mean ± standard deviation. Different lowercase letters within each column indicate significant differences among treatments according to false discovery rate-adjusted *post-hoc* comparisons at p ≤ 0.05.

### 3.3 Correlation matrix

Pearson correlation analysis was conducted to examine the relationships among the priming solutions as represented in **Table 3**. The analysis revealed significant positive correlations among most variables. Strong correlations were observed between K and C (r, 0.816, p< 0.001), C and W (r, 0.832, p< 0.001), and PEG and I (r, 0.731, p< 0.01). Moderate correlations were detected between K and W (r, 0.786, p< 0.001), K and PEG (r, 0.680, p< 0.01), C and PEG (r, 0.698, p< 0.01), K and I (r, 0.495, p< 0.05), and C and I (r, 0.510, p< 0.05). A weak, non-significant correlation was observed between W and I (r, 0.437, p ≥ 0.05). Overall, these results indicate that K, C, and W are closely associated, while I is more strongly associated with PEG than with other variables. Pearson correlation coefficients among priming treatments ranged from r, 0.437 to r, 0.832, with the highest correlation observed between C and W (r, 0.832) and the lowest between W and I (r, 0.437). The significant correlations among several priming treatments correspond with the overall significant multivariate treatment effects identified by MANOVA (Wilks’ Λ, 0.177, p< 0.001) (**Table 1**). To assess robustness of the correlation structure, Spearman’s rank correlation analysis was additionally performed. The overall correlation patterns were consistent with Pearson correlation results.

**Table 3:** Pearson correlation analysis presenting the relationship among the priming solutions.

	K	C	W	PEG	FeSO_4_
K		0.816**	0.786**	0.680*	0.495*
C			0.832**	0.698*	0.510*
W				0.673*	0.437^ns^
PEG					0.731*

W, distilled water; FeSO_4_, iron sulphate; PEG, polyethylene glycol; K, kinetin; C, control. Bars with the same letters are not significantly different (p > 0.05). **Asterisks indicate significant differences: *p< 0.05, **p< 0.01, ***p< 0.001, ns, not significant.

### 3.4 Antioxidant activity of the different plant extracts

**Figure 2:**
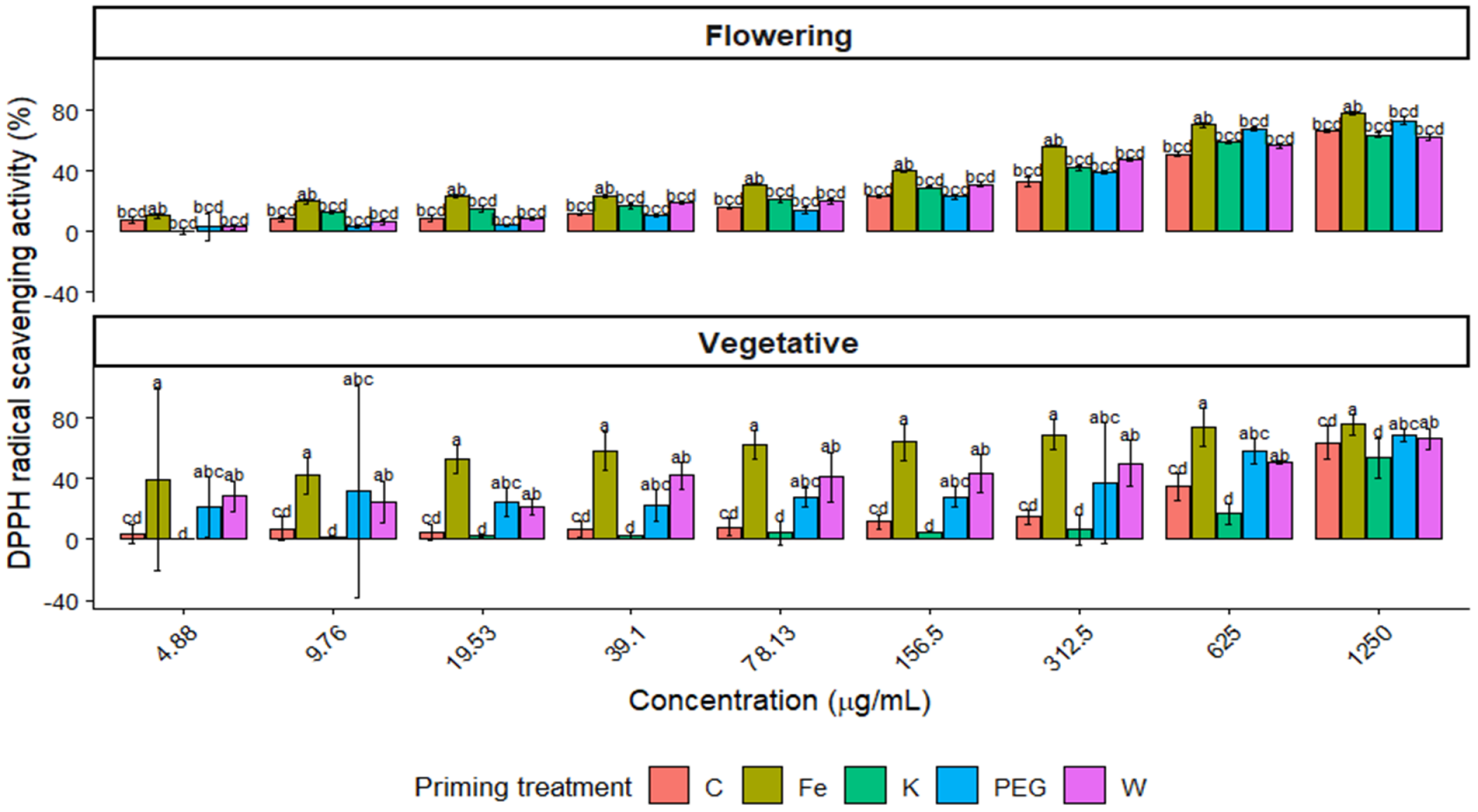
DPPH radical scavenging activity (%) of *Corchorus trilocularis* extracts obtained from vegetative and flowering stages following seed priming with different solutions [kinetin (K); polyethylene glycol (PEG); iron sulphate (FeSO_4_); water (W); untreated control (C)] across increasing extract concentrations (4.88-1250 µg/mL). Values represent mean ± standard deviation of three biological replicates (n, 3). Error bars indicate standard deviations. Statistical differences among priming treatments within the same concentration and developmental stage were determined using analysis of variance (ANOVA) followed by Tukey’s honestly significant difference (HSD) *post-hoc* test at p< 0.05. Bars sharing the same letters within a concentration and developmental stage are not significantly different. Ascorbic acid was included as the positive control for the DPPH assay.

**Figure 3:**
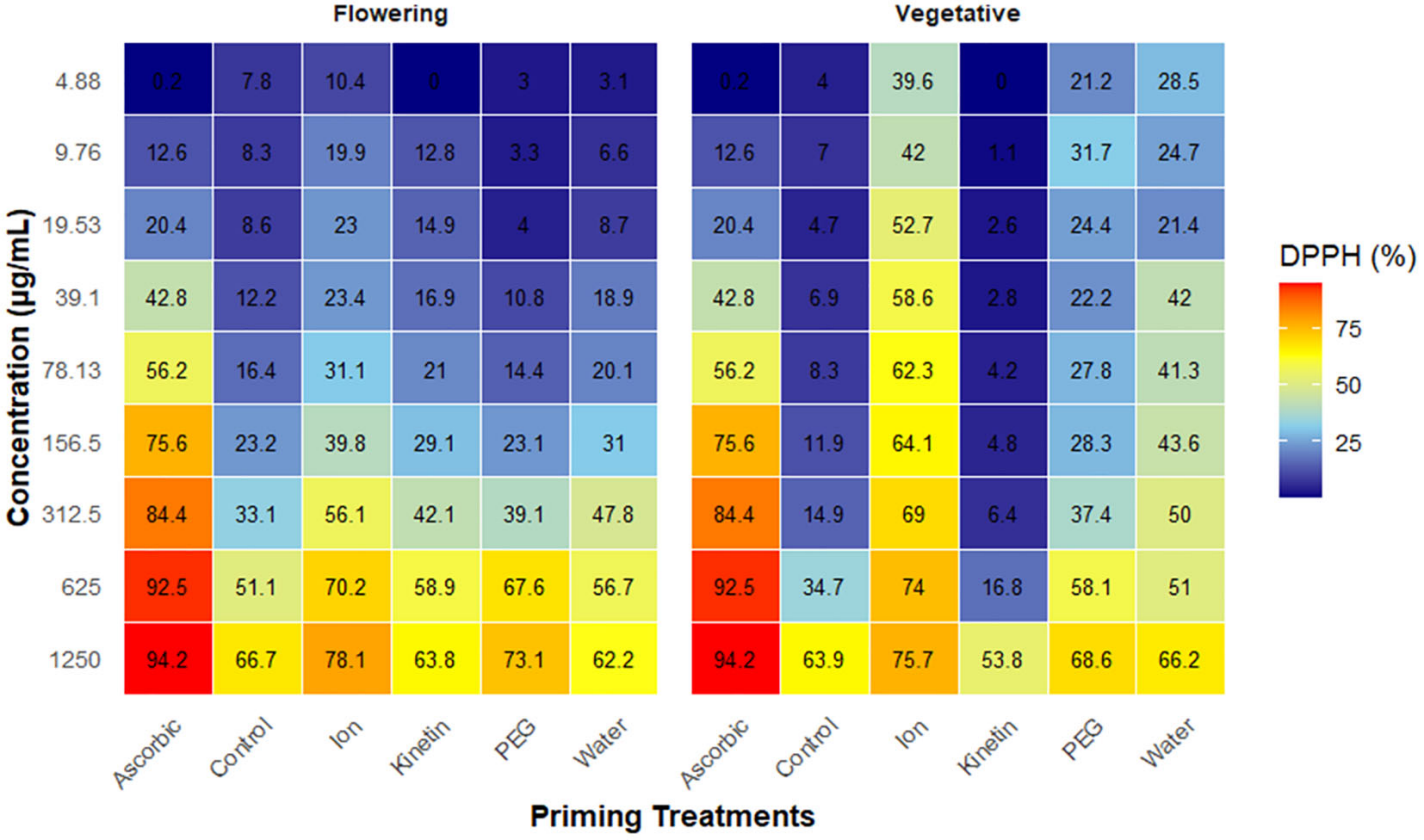
Heatmap illustrating DPPH radical scavenging activity (%) of *C. trilocularis* under different priming treatments across a range of concentrations (μg/mL). The two panels represent the vegetative stage (left) and flowering stage (right). Colour intensity reflects the magnitude of antioxidant activity, with a gradient from blue (low activity) to red (high activity). Numerical values within each cell indicate the exact DPPH (%) values, enabling direct quantitative comparison across treatments and concentrations. priming treatments include ascorbic acid, control, FeSO_4_, kinetin, PEG, and water.

Bar graphs depicting DPPH radical scavenging activity (DPPH, %) across priming solutions and concentrations revealed distinct patterns between vegetative and flowering stages (**Figure 2**). Across most concentrations, FeSO_4_ and PEG consistently exhibited the highest DPPH scavenging potentials, particularly at higher concentrations (≥312.5 μg/mL). In the vegetative stage, FeSO_4_ and PEG were the most effective, while kinetin generally showed lower DPPH across concentrations. Overall, DPPH increased with concentration, demonstrating a clear dose-response relationship, and stage-specific differences were apparent, with flowering stage showing higher antioxidant activity for different effective treatments. The heatmap facilitated rapid identification of the most effective priming solutions and revealed distinct dose - response patterns, as well as stage-specific differences, with flowering stage exhibiting higher antioxidant activity for certain treatments (**Figure 3**). Ascorbic acid served as the positive control for the DPPH assay and is shown as a common reference for both vegetative and flowering stages, as the assays were performed concurrently.

### 3.5 Minimum inhibitory concentration and minimum bactericidal concentration of the different extracts against *S. aureus* and *P. aeruginosa*

At flowering stage, the extracts demonstrated variable inhibitory activity against *S. aureus* depending on the priming treatment (**Table 4**). The PEG-primed treatment exhibited the strongest antimicrobial activity, followed by the I primed treatment, while the control treatment generally showed the lowest activity. The lowest MIC values were observed in the PEG-primed extracts (0.14 mg/mL). This was followed by the iron-primed treatment (0.16 mg/mL). In contrast, the control treatment showed the highest MIC value (0.79 mg/mL). Extracts from the K and W priming treatments displayed intermediate MIC values of 0.58 mg/mL and 0.63 mg/mL, respectively. A similar pattern was observed for MBC. The PEG treatment demonstrated the most potent bactericidal activity with an MBC of 1.18 mg/mL, closely followed by the I treatment with an MBC of 1.25 mg/mL. In contrast, extracts from the K, C, and W treatments showed weaker bactericidal effects with MBC values reported as ≤2.5 mg/mL.

**Table 4:** Minimum inhibitory and bactericidal concentration, and total activity of the leaf extract from different priming solution tested against *Staphylococcus aureus* and *Pseudomonas aeruginosa*.

				Priming solutions												
Bacteria and control	K			C			W			PEG				FeSO_4_		
Flowering Stage																
	MIC	MBC	TA	MIC	MBC	TA	MIC	MBC	TA		MIC	MBC	TA	MIC	MBC	TA
S. *aureus*	0.58± 0.35	2.5± 0.07	334.83± 0.12	0.79± 0.09	< 2.5	254.43± 0.09	0.63± 0.02	< 2.5	317.46± 0.02		0.14± 0.1	1.18± 0.12	1428.57± 0.09	0.16± 0.20	1.25± 0.02	1250.00± 0.11
P.*aeruginosa*	–	–	–	–	–	–	–	–	–		–	–	–	–	–	–
Ampicillin		0.05± 0.01														
Vegetative Stage																
S. *aureus*	–	–	–	–	–	–	–	–	–		–	–	–	–	–	–
P. *aeruginosa*	–	–	–	–	–	–	–	–	–		–	–	–	–	–	–
Ampicillin		0.05± 0.01														

MIC and MBC values are presented as mean ± standard deviation of three biological replicates. MIC, minimum inhibitory concentration; MBC, minimum bactericidal concentration; TA, total activity; K, kinetin; C, control; W, water; PEG, polyethylene glycol; FeSO_4_, iron sulphate. MIC and MBC determinations were conducted using three technical and three biological replicates, and reported values represent the predominant consensus concentrations observed across replicates. Minor variation between replicates was observed due to the discrete dilution-based nature of serial dilution antimicrobial assays. “-” indicates no detectable antibacterial activity at the concentrations tested. Vegetative-stage extracts showed no detectable antibacterial activity and were therefore excluded for clarity.

The PEG treatment exhibited the highest total activity value (1428.57 mL/g). The I treatment also showed a high total activity value (1250.00 mL/g), reflecting strong antimicrobial potential. In contrast, K, W and C treatments showed considerably lower TA values of 334.83 mL/g, 317.46 mL/g and 254.43 mL/g, respectively. Conversely, the control treatment produced extracts with the weakest antimicrobial activity. No inhibitory or bactericidal activity was observed against *P. aeruginosa* for any of the treatments at the tested concentrations during the flowering stage. Similarly, extracts from the vegetative growth stage did not produce any inhibitory or bactericidal activity against *S. aureus* and *P. aeruginosa*.

### 3.6 Growth kinetics

#### 3.6.1 Two-way ANOVA

Two-way analysis of variance (ANOVA) was performed to determine the effects of priming solution, incubation time, and their interaction on *C. trilocularis* growth (**Table 5**). The analysis revealed that both priming treatment and time significantly affected OD600 values.

**Table 5:** Analysis of variance of mean squares for the effects of priming treatments, incubation time, and their interaction on the growth kinetics of *S. aureus* in the presence of *C. trilocularis* extracts.

Source of variation	Df	Sum of Squares	Mean squares	F-value	P-value
Priming Solution (PS)	5	17.41	1.65	313.30	< 2.0 × 10^-^¹^6^ ***
Time	10	34.49	13.24	3724.00	< 2.0 × 10^-^¹^6^ **
PS × Time	50	14.02	1.54	252.30	< 2.0 × 10^-^¹^6^ ***
Residual	217	2.01	0.014		

Df, degrees of freedom; PS, priming solution; *** significant at p< 0.001.

#### 3.6.2 Impact of priming solution on growth dynamics of *Staphylococcus aureus*

**Figure 4:**
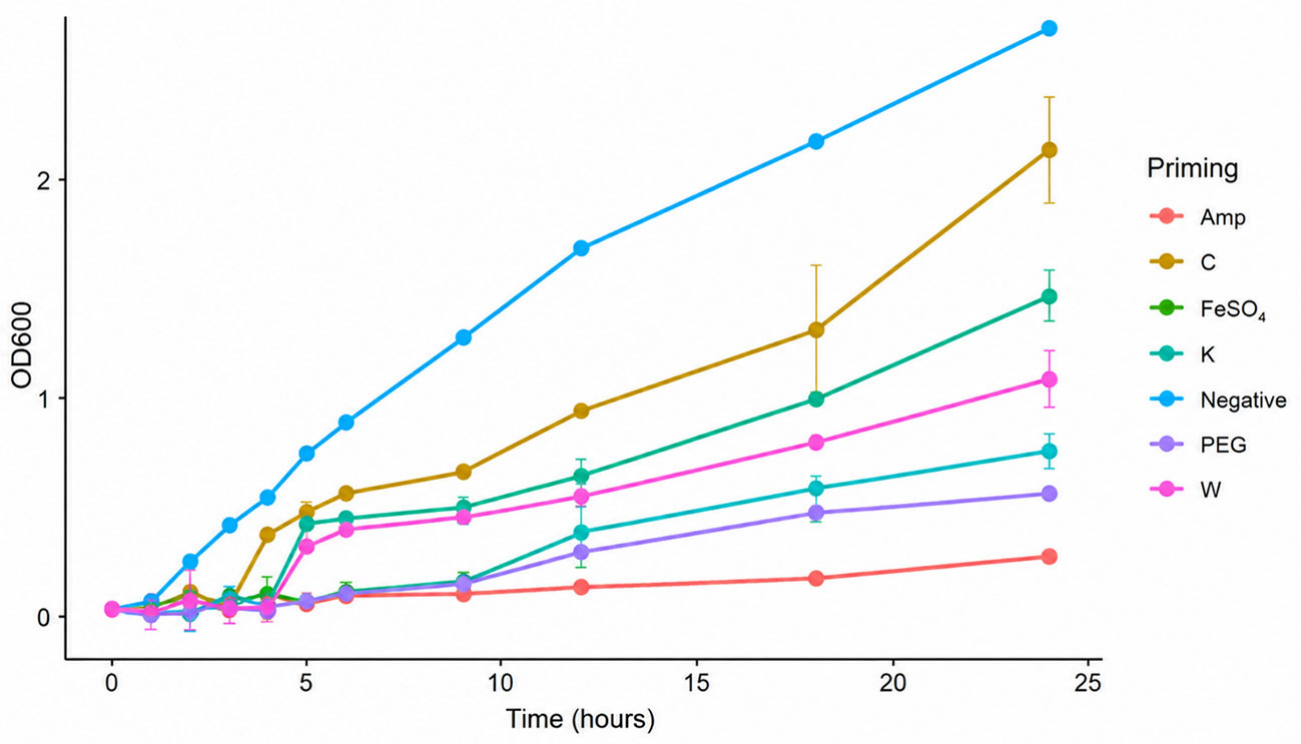
The effect of extracts from the flowering growth stage primed with different solutions on the growth of *S. aureus* over a 24-hour period. Amp, ampicillin; C, control (non-primmed extracts); I, ion sulphate; Negative, negative control (bacterial culture with no extracts); PEG, polyethylene glycol; W, water.

Since antibacterial activity was observed only against *S. aureus*, this bacterium was subsequently used to evaluate the effects of the extracts on bacterial growth kinetics. Distinct growth patterns of *S. aureus* were observed among different priming solutions from the flowering stage (**Figure 4**). During the initial incubation period (0–4 h), minimal increases in OD600 were observed across most treatments. A pronounced increase in growth rate became evident between 5 and 9 h. The most substantial increases in OD600 occurred between 9 and 18 h, particularly in the negative control, K, W, and C treatments. Beyond 18 h, growth began to plateau in several treatments. The negative control exhibited the highest growth throughout the incubation period, with a marked increase beginning at 6–9 h and continuing rapidly until 18 h, ultimately reaching a maximum OD600 of 2.69 at 24 h. In contrast, the antibiotic control (Amp) strongly inhibited bacterial growth, with OD600 remaining relatively stable throughout the experiment and reaching only 0.26 at 24 h. Among the priming treatments, C treatment supported the highest bacterial growth, with a sharp increase in OD600 observed between 6 and 12 h, followed by continued growth until 24 h, reaching a maximum OD600 of 2.41. The K treatment also showed a pronounced exponential growth phase between 5 and 12 h, resulting in a final OD600 of 1.53, while W treatment exhibited moderate growth acceleration beginning at approximately 6 h, reaching 1.24 OD600 at 24 h. Moderate growth was observed for the I treatment, with gradual increases occurring primarily between 9 and 18 h, resulting in a final OD600 of 0.94. In comparison, PEG treatment displayed slower growth kinetics, with only modest increases occurring after 9 h, and reached a maximum OD600 of 0.55.

#### 3.6.3 Tukey’s honest significant difference *post hoc* analysis

Tukey’s Honest Significant Difference (HSD) *post hoc* analysis revealed that treatment effects were not uniform across incubation time but instead exhibited a clear time-dependent pattern (**Table 6**). At early stages (0–4 h), no significant differences were observed among treatments. However, from 5–6 h onwards, divergence between treatments began to emerge, with significant differences (p< 0.01) becoming evident between control and primed treatments. The most pronounced separation occurred at 12–24 h, where Amp (ΔOD600, -0.858, p< 0.0001), K (ΔOD600, -0.549, p< 0.0001), W (ΔOD600 ≈ -0.620, p< 0.0001), and other plant-based treatments significantly reduced bacterial growth compared to the negative control.

**Table 6:** .

Time (h)	Comparison	Estimate (ΔOD600)	P-value	Interpretation
0	All treatments	~0	ns	No initial differences
1-4	Most comparisons	small	>0.05	Early lag phase, minimal separation
5-6	K vs Neg	↓ significant	<0.001	Onset of divergence
9	PEG vs C	moderate difference	<0.01	Treatment effects emerging
12	Amp vs Neg	large reduction	<0.0001	Strong inhibition begins
18	All treatments vs Neg	large negative estimates	<0.0001	Strong antimicrobial activity
24	Amp/K/C vs Neg	strongest effects	<0.0001	Maximum treatment separation

### 3.7 Extracts combinational effects

At flowering stage, the combinational antibacterial activity of *C. trilocularis* extracts from different seed priming treatments was evaluated against *S. aureus* using fractional inhibitory concentration (FIC) analysis calculated using Equation 1 and Equation 2 (**Table 7**). Across all combinations tested at the flowering stage, ΣFIC values ranged from 14.27 to 36.27. The highest antagonism was observed in the C + PEG combination (ΣFIC, 36.27), while the lowest was observed in C + W (ΣFIC, 14.27). No combinational activity was observed against *Pseudomonas aeruginosa*, consistent with the lack of antibacterial activity of individual extracts against this organism. At vegetative stage, no combinational activity was observed against *S. aureus* and *P. aeruginosa*, consistent with the lack of antibacterial activity of individual extracts against these organisms.

**Table 7:** Combinational antibacterial activity of primed *C. trilocularis* extracts against *S. aureus*.

Combinations	FIC (A)	FIC (B)	FIC index (ΣFIC)	Outcome
Flowering Stage				
K + C	8.62 ± 2.30	6.33 ± 0.00	14.9 ± 2.09	antagonistic
K + W	8.62 ± 1.95	7.94± 2.00	16.56 ± 2.35	antagonistic
K + PEG	6.47 ± 2.22	26.78± 6.08	33.24 ± 6.66	antagonistic
K + I	6.47± 2.62	23.44± 2.65	29.91± 7.02	antagonistic
C + W	6.33± 3.03	7.94± 1.23	14.27± 3.03	antagonistic
C + PEG	9.49 ± 0.00	26.78± 3.62	36.27± 4.38	antagonistic
C + I	4.93± 2.59	23.44± 7.32	28.37± 6.57	antagonistic
W + PEG	5.95± 0.95	26.78± 7.11	32.38± 4.95	antagonistic
W + I	5.95± 1.11	23.44± 6.35	29.39± 9.21	antagonistic
PEG + I	17.80± 4.06	15.63± 3.22	33.43± 6.25	antagonistic

FIC, Fractional Inhibitory Concentration; ΣFIC, sum of individual FIC values for each treatment combination. Interaction categories were defined as follows: synergy (ΣFIC ≤ 0.5), additive (<no>0.5<</no> ΣFIC ≤ 1), indifferent (<no>1<</no> ΣFIC ≤ 4), and antagonistic (ΣFIC > 4), indicating strong inhibitory opposition between treatments. Values are presented as mean ± standard deviation from biological replicates. “C” represents control (extracts from non-primed plants). .

## 4 Discussion

The present study indicates that seed priming and plant developmental stages are associated with treatment-dependent phytochemical and bioactivity changes in *Corchorus trilocularis*, with potential implications for antimicrobial efficacy. The study was conducted using three biological replicates with technical replication to improve measurement reliability. Although this replication level is consistent with many controlled plant physiology and phytochemical studies, larger sample sizes and formal *a priori* power analyses would further strengthen statistical precision and generalisability of the findings. The multivariate analysis showed that both seed priming treatment and growth stage had highly significant multivariate effects on phytochemical accumulation (p< 0.001), with a significant interaction between these factors (**Table 1**). This interaction indicates that the effectiveness of priming treatments is developmentally regulated rather than constant across growth stages, reflecting the dynamic nature of plant secondary metabolism.

The phytochemical analyses performed in this study focused on broad quantitative classes of secondary metabolites rather than compound-specific identification. Therefore, the observed biological activities cannot be attributed to individual phytochemical constituents. Advanced analytical approaches such as HPLC-UV, LC-MS/MS, and metabolomic profiling would provide improved resolution of the specific compounds associated with the observed bioactivities. Flavonoid and phenolic contents were significantly higher at the flowering stage across treatments, reflecting a developmental shift towards enhanced secondary metabolism (**Figure 1**). However, the response was phytochemical- and developmental stage-dependent rather than universally superior across all measured parameters. For example, polyethylene glycol and FeSO_4_ produced comparable flavonoid responses during the vegetative stage, where differences between the two treatments were not statistically significant. These findings suggest that the effectiveness of priming agents depends on both the specific class of secondary metabolites evaluated and the developmental stage of the plant. Several researchers have reported similar effects of growth stage on the phytochemical content and antioxidant activities in different crops including *Celosia argentea L.* (Adegbaju et al., 2020), strawberry cultivars and mulberry (Mahmood et al., 2012) and *Agastache rugosa* (Hwang et al., 2024). Therefore, the novelty of the present study does not lie in the observation of increased secondary metabolite accumulation during flowering itself, but rather in demonstrating how this developmental increase interacts with seed priming treatments and relates to antioxidant and antibacterial activities in *C. trilocularis*.

The findings in this study suggest that the flowering stage may represent a physiologically favourable period for maximising the functional bioactivity of primed plants. Based on previous studies, the increased accumulation of flavonoids and phenolics may be associated with the plant’s requirement for heightened defence during reproduction, where secondary metabolites play protective roles against oxidative stress and microbial attack (Solouki et al., 2023). Among the treatments, FeSO_4_priming exerted the most pronounced and consistent effect on phytochemical accumulation, maintaining elevated phenolic levels across both vegetative and flowering stages. Previous studies have associated iron availability with redox-related metabolic processes and phenylpropanoid metabolism (Wasli et al., 2021; Du et al., 2023; Ziaei et al., 2025). Therefore, the enhanced accumulation of phenolics and flavonoids observed in the present study may be associated with iron-mediated modulation of secondary metabolic pathways. However, enzymatic activity and molecular mechanisms, including the involvement of enzymes such as phenylalanine ammonia-lyase, were not directly evaluated and therefore remain speculative in this study. Furthermore, the phytochemical data should be interpreted at the level of broad secondary metabolite shifts, encompassing total phenolic pool changes and flavonoid class accumulation, without inferring compound-specific activity or direct activation of biosynthetic pathways, as these were not experimentally assessed.

Iron-mediated modulation of reactive oxygen species has previously been associated with signalling processes involved in secondary metabolite accumulation (Ali et al., 2022). The comparatively high phenolic content observed under FeSO_4_treatment, even during the vegetative stage, may therefore reflect altered metabolic responses associated with iron availability (**Figure 2**). However, reactive oxygen species signalling and associated metabolic pathways were not directly quantified in the present study. These are similar impacts to those observed in *Brassica juncea* L. in which ferrous sulphate enhanced the effects of thylakoidal multiprotein complexes, metabolism and defence under arsenic stress (Ali et al., 2022).

Polyethylene glycol priming also significantly enhanced phytochemical accumulation, particularly during the flowering stage. With a more pronounced effect observed during the flowering stage, indicating a strong growth stage-dependent response. This suggests that developmental status modulates the extent to which osmotic priming influences secondary metabolite accumulation. Polyethylene glycol-induced osmotic stress has previously been reported to stimulate oxidative stress-related responses and antioxidant-associated metabolic pathways (Sheteiwy et al., 2016; Ma et al., 2024). The increased flavonoid and phenolic accumulation observed in the present study may therefore reflect stress-associated metabolic adjustments; however, antioxidant enzyme activity and signalling pathways were not directly assessed. The relatively strong association between polyethylene glycol and FeSO_4_ observed in the correlation matrix (r, 0.731, p< 0.01) further supports a shared biochemical outcome, despite differing mechanisms of action.

Although kinetin is a cytokinin commonly associated with enhanced cell division and biomass accumulation, its effects on secondary metabolism are often variable and species-dependent (Grzegorczyk-Karolak et al., 2015). Previous studies have indicated that cytokinins may preferentially promote primary growth processes rather than the allocation of resources towards secondary metabolite biosynthesis, particularly under non-stress conditions (Emery and Kisiala, 2020). This may partly explain the comparatively moderate phytochemical responses observed under kinetin treatment in the present study. Further, previous studies have similarly reported that enhanced vegetative growth does not necessarily correspond with increased secondary metabolite accumulation, highlighting the complex balance between primary and secondary metabolism in plants (Hout et al 2014).

Tannin accumulation showed comparatively lower treatment differentiation than flavonoids and phenolics across priming treatments. This may indicate that tannin biosynthesis in *C. trilocularis* is more tightly developmentally regulated or constitutively maintained and therefore less responsive to external priming stimuli (Mora et al., 2022). Tannin synthesis is also often associated with higher metabolic costs and stricter genetic regulation, with accumulation preferentially induced under specific developmental or stress conditions rather than maintained at continuously high levels. In contrast, flavonoids and simple phenolics may exhibit more dynamic responses to priming treatments due to their broader roles in antioxidant defence and stress-related signalling pathways.

The false discovery rate-adjusted *post-hoc* comparisons strengthened the reliability of the observed treatment differences by controlling for Type I error associated with multiple pairwise comparisons. Even after adjustment, FeSO_4_ priming consistently maintained significantly greater phenolic and flavonoid accumulation, particularly during the flowering stage. Polyethylene glycol also promoted moderate phytochemical enhancement, although its effects were generally less pronounced than those observed for FeSO_4_priming. In contrast, kinetin, water, and the control treatments frequently grouped within lower statistical classes. The false discovery rate correction indicates that the observed responses were biologically meaningful rather than statistical artefacts arising from repeated comparisons.

The relatively large standard deviations observed for several treatments, reflect inherent biological variability in secondary metabolite accumulation, which is expected in plant systems influenced by developmental stage and microenvironmental heterogeneity. Replication across biological and technical replicates ensured robustness of the observed statistical trends.” particularly during the flowering stage, indicate considerable biological variability in metabolite accumulation, which may reflect heterogeneous physiological responses to priming and environmental interactions. Despite this variability, the consistent statistical groupings suggest that the observed treatment effects are robust and reproducible across growth stages, reinforcing the reliability of the trends reported.

The strong positive correlations provided by the Pearson correlation analysis between kinetin, control, and water indicate that these treatments may produce similar phytochemical responses, likely reflecting baseline metabolic activity with limited biochemical stimulation (**Table 3**). This suggests that kinetin, under the conditions tested, does not substantially deviate from natural physiological processes in terms of secondary metabolite induction. In contrast, the weaker correlations between FeSO_4_ and the other treatments highlight its potential distinct mode of action, reinforcing its role as a strong modulator of plant metabolism and supporting the comparatively greater phytochemical accumulation observed under FeSO_4_ priming. The moderate correlations between polyethylene glycol and other treatments suggest partial overlap, consistent with its ability to induce stress-related pathways that complement but do not fully replicate iron-mediated effects. The correlation structure suggests that certain priming treatments, particularly control and water, exhibit closely aligned phytochemical response profiles, whereas FeSO_4_ shows greater divergence relative to baseline treatments. When considered alongside the significant multivariate effects observed in the MANOVA, these patterns indicate that treatment differences are expressed not only as overall statistical separation but also as varying degrees of similarity within the multivariate response space. The consistency between Pearson and Spearman correlation analyses confirms that the observed relationships are robust despite deviations from normal distributional assumptions.

Antioxidant activity, as measured by 2,2-diphenyl-1-picrylhydrazyl radical scavenging, closely mirrored the patterns observed for phenolic and flavonoid accumulation, providing functional validation of the phytochemical data. Iron sulphate and polyethylene glycol treatments consistently exhibited the highest radical scavenging activity, particularly at higher extract concentrations and during the flowering stage (**Figures 2**, **3**). Phenolics and flavonoids are well-established antioxidant compounds due to their ability to donate electrons or hydrogen atoms and neutralise reactive oxygen species (Dai and Mumper, 2010). Similarly, Liang et al. (2026), reported that seed priming improves plant tolerance to stress by activating antioxidant pathways and enhancing metabolic efficiency. Therefore, the comparatively higher antioxidant activity observed in the current study in FeSO_4_ - and PEG-primed plants may be associated with the increased accumulation of these antioxidant-related secondary metabolites. The enhanced antioxidant activity observed following PEG and FeSO_4_ priming may be associated with treatment-induced modulation of secondary metabolism and antioxidant defence responses. PEG-mediated osmotic stress has previously been reported to stimulate the accumulation of protective antioxidant compounds under controlled stress conditions, while iron plays important roles in redox regulation and enzymatic processes associated with phenolic metabolism and antioxidant activity (Saleem et al., 2022; Du et al., 2023; Ma et al., 2024). The comparatively higher DPPH scavenging activity observed in flowering-stage extracts may therefore reflect developmental increases in secondary metabolite accumulation combined with treatment-associated biochemical responses. However, the present study did not directly evaluate enzymatic activity, reactive oxygen species signalling, or molecular pathways; therefore, these interpretations remain associative rather than mechanistically confirmed. Also, Although the DPPH assay provided useful comparative insight into radical scavenging activity, antioxidant capacity is multifaceted and cannot be fully represented by a single assay. Complementary methods such as ABTS, FRAP, and ORAC would provide a broader assessment of antioxidant potential. Overall the alignment observed in the present study between phytochemical content and antioxidant activity further supports the biochemical linkage between priming-induced metabolic enhancement and functional bioactivity.

Antibacterial activity was primarily observed against the Gram-positive bacterium *Staphylococcus aureus*, particularly in flowering-stage extracts, whereas no detectable activity was observed against the Gram-negative *Pseudomonas aeruginosa*. Polyethylene glycol and FeSO_4_ priming were associated with increased *in vitro* antibacterial activity, as evidenced by lower minimum inhibitory concentration and minimum bactericidal concentration values compared to the control (**Table 4**). This enhanced efficacy may be attributed to elevated phenolic and flavonoid contents, which are known to disrupt bacterial membranes, increase permeability, and interfere with intracellular targets such as enzymes and nucleic acids (Nazzaro et al., 2013). However, although certain extracts inhibited bacterial growth at lower concentrations, the MBC values reported as “> 2.5 mg/mL” indicate that complete bactericidal activity was not achieved within the concentration range tested. This suggests that the extracts exhibited bacteriostatic effects than bactericidal effects under the conditions evaluated, and that higher concentrations may be required to achieve complete bacterial killing.

These antimicrobial effects were pronounced against *S. aureus*, a Gram-positive bacterium with a relatively permeable cell wall, whereas no detectable activity was observed against *P. aeruginosa*. This may partly reflect the inherent tolerance commonly associated with Gram-negative bacteria, including the presence of an outer membrane that can limit the penetration of antimicrobial compounds (Cushnie and Lamb, 2011). *Staphylococcus aureus*, a Gram-positive bacterium, lacks an outer membrane, allowing easier penetration of bioactive phytochemicals such as phenolics and flavonoids. In contrast, *P. aeruginosa*, a Gram-negative bacterium, possesses an outer membrane composed of lipopolysaccharides, which restricts the entry of antimicrobial compounds. Additionally, *P. aeruginosa* exhibits intrinsic resistance mechanisms, including efflux pumps, which further reduce the effectiveness of plant-derived compounds. The selective antibacterial activity of *C. trilocularis* extracts also has implications in the context of antimicrobial resistance. *Staphylococcus aureus* is a clinically important pathogen frequently associated with multidrug-resistant infections, while Pseudomonas aeruginosa is recognised as a critical priority pathogen due to its intrinsic resistance mechanisms and ability to withstand multiple classes of antibiotics. The observed inhibitory effects against *S. aureus* suggest that *C. trilocularis* contains bioactive compounds capable of interfering with essential bacterial processes, potentially through membrane disruption and metabolic inhibition. In contrast, the lack of activity against *P. aeruginosa* reflects its highly effective outer membrane barrier and efflux systems, which limit intracellular accumulation of phytochemicals. Importantly, plant-derived phytochemicals typically act on multiple cellular targets, reducing the likelihood of rapid resistance development and highlighting their potential as complementary sources for future antimicrobial discovery. However, membrane permeability and compound uptake were not directly assessed in the present study. Additionally, the absence of activity may also be related to insufficient concentrations of active phytochemicals or the lack of compounds specifically effective against *P. aeruginosa*. Therefore, the precise factors underlying the observed resistance remain unresolved and require further investigation using compound-specific and mechanistic approaches. Approaches. reflecting the protective outer membrane and efflux mechanisms characteristic of Gram-negative bacteria (Cushnie and Lamb, 2011), although these mechanisms were not directly assessed in this study. The antibacterial assessment in the present study was limited to two bacterial strains under *in vitro* conditions; therefore, the findings should not be interpreted as evidence of broad-spectrum antimicrobial activity.

Furthermore, the absence of antimicrobial activity at the vegetative stage (**Table 4**) highlights the importance of developmental stage, as phytochemical concentrations at this stage are likely insufficient to achieve effective antimicrobial action, consistent with the lower antioxidant activity observed. Total activity values further supported the antimicrobial potential of polyethylene glycol and FeSO_4_ treatments by integrating extract yield with antimicrobial efficacy, thereby providing a broader assessment of bioactivity beyond minimum inhibitory concentration values alone. The total activity values obtained in this study are comparable to those previously reported for plant-derived antimicrobial extracts, where total activity is commonly used as a comparative efficiency indicator across species and extraction systems, with higher values reflecting greater antimicrobial potency per unit plant material (Vaisbourd et al., 2025). Total activity values for PEG and FeSO_4_ treatments exceeded 1000 mL/g, indicating exceptionally high antimicrobial efficiency relative to plant biomass, and falling within the upper range of values reported for highly active medicinal plant extracts in Eloff-type antimicrobial screening frameworks. In contrast, kinetin, control, and water treatments showed moderate total activity values consistent with lower antimicrobial efficiency (Eloff, 1998).

In the present study, control and untreated groups exhibited a rapid increase in OD600 over time, reflecting unrestricted bacterial proliferation. In contrast, priming treatments such as PEG and I consistently reduced bacterial growth rates across the incubation period, particularly during the early exponential phase. This interaction effect indicates that treatment differences became increasingly pronounced at later time points, where control samples showed exponential growth while treated samples maintained comparatively lower OD values. The delayed exponential phase and reduced maximum cell density under polyethylene glycol treatment, in particular, indicate bacteriostatic effects with potentially bactericidal activity supported by BMC results (**Figure 4**) (Udekwu et al., 2009; Rolfe et al., 2012; Vaisbourd et al., 2025). In contrast, extracts from control, kinetin, and water treatments allowed rapid bacterial proliferation, reflecting their lower phytochemical content and weaker antimicrobial activity. The gradual rather than immediate inhibition pattern from polyethylene glycol and iron treatments suggests that these extracts may exert cumulative effects, progressively impairing bacterial growth over time. These observations are similar to the reported antibacterial potentials of *Lamiaceae* plant extracts that significantly reduced *Pseudomonas aeruginosa* PAO1 invasion (alđe Pavlović et al., 2024). Similarly, antimicrobial activity and phytochemical profiling of natural plant extracts for biological control of wash water in the agri-food industry presented similar inhibition patterns (Kanarek et al., 2025). Further, a highly significant interaction between priming solution and incubation time (PS × Time, F, 252.30, p< 0.001) indicates that the effect of priming treatments on bacterial growth was strongly time-dependent. This demonstrates that bacterial growth kinetics were not uniform across treatments but instead followed distinct temporal response patterns (**Table 5**). Pairwise comparisons using Tukey’s Honest Significant Difference test further confirmed that the treatment × time interaction reflects a dynamic, stage-dependent antimicrobial response rather than a constant effect across the incubation period (**Table 6**). This is consistent with the mode of action of many plant-derived phenolics, which often target multiple cellular pathways simultaneously. These findings highlight the importance of linking biochemical composition with biological activity and demonstrate that targeted agronomic interventions can improve the bioactivity profile of medicinal plants; however, antimicrobial effects appear to be selective and dependent on bacterial species and structural characteristics. The antagonistic interactions observed among the combined extracts have been previously reported in previous studies investigating interactions among herbal and plant-derived extracts, where reduced bioactivity was observed in combined formulations. This include studies that monitored the effects of some commonly used herbs on the efficacy of Artemisinin derivatives in the treatment of malaria in experimental mice (Idowu et al., 2020) and infusions of green tea with bee honey and *Citrus limonum* extract as additives (Uduwana et al., 2023). The antagonistic interactions observed in the present study can be attributed to several underlying biochemical properties associated with the priming treatments (**Table 7**). For example, (FeSO_4_), due to its redox-active nature, may potentially interact with phenolic and flavonoid compounds through metal chelation, thereby reducing the availability of these bioactive molecules for antimicrobial action (Kejík et al., 2021; Mussio et al., 2025). Polyethylene glycol priming enhances the accumulation of antioxidant compounds, which may counteract iron-induced reactive oxygen species, influence oxidative balance and could indirectly affect antimicrobial activity when combined with other extracts (Caesar and Cech, 2019). Additionally, phenolics and flavonoids can compete for binding sites on bacterial cell walls or interact with each other through hydrogen bonding and hydrophobic interactions, reducing their overall effectiveness. Based on previous studies, the presence of tannins further contributes to antagonism by forming insoluble complexes with proteins and other phytochemicals, limiting their diffusion and bioavailability (Soares et al., 2020). Importantly, these antagonistic interactions predominantly occur in combined extracts because each individual extract may contain phytochemicals in ratios that minimize internal interference, whereas mixing extracts introduces imbalanced ratios and new cross-interactions that reduce the availability of free, active compounds. In addition, variability among FIC estimates was high due to the very low MIC values obtained for extracts alone compared to in combination, which mathematically amplified the FIC ratios. Collectively, these interactions may reduce the concentration or accessibility of active antimicrobial compounds, contributing to the observed antagonistic effects. However, the precise biochemical basis of these antagonistic interactions remains unresolved and the mentioned mechanisms were not directly evaluated in the present study and therefore remain speculative.

## 5 Conclusions

Seed priming and plant developmental stage jointly regulate phytochemical accumulation in *C. trilocularis*, with effects that vary dynamically across growth stages and significantly influence antioxidant and *in vitro* antimicrobial potential. The flowering stage consistently promoted higher flavonoid and phenolic content, while FeSO_4_ and polyethylene glycol priming were the most effective in enhancing secondary metabolite production, potentially through physiological and metabolic responses previously associated with enzymatic and stress-related pathways, although these mechanisms were not directly assessed in this study. These biochemical enhancements translated into increased antioxidant activity and improved antibacterial efficacy, particularly against *S. aureus*, under the conditions tested. Thus highlighting the functional relevance of priming-associated metabolic changes under *in vitro* conditions. The absence of activity during the vegetative stage and against *P. aeruginosa* underscores the importance of both sufficient metabolite accumulation and bacterial structural differences in determining antimicrobial outcomes. However, the observed antagonistic interactions in combined extracts emphasize that phytochemical balance is critical, as mixing extracts can disrupt optimized ratios and reduce overall bioactivity, reinforcing the need for targeted and strategic application of priming -negative pathogens, as well as clinically relevant multidrug-resistant isolates. Future investigations should include extended bacterial growth kinetics analyses beyond the 24 h incubation period, together with colony-forming unit enumeration and additional physiological assessments, to better characterise bacterial adaptation, survival responses, and long-term effects of the extracts under antimicrobial stress conditions. Furthermore, advanced phytochemical profiling techniques such as liquid chromatography-tandem mass spectrometry and *in vivo* studies are needed to better elucidate the specific bioactive compounds and validate their therapeutic potential. Future studies should also include *in vivo* pharmacological and toxicity evaluations to validate the biological relevance of these findings under physiological conditions treatments. Importantly, all antimicrobial assays were conducted *in vitro*; therefore, no *in vivo* or clinical efficacy can be inferred from these results.

Although the present study demonstrated clear associations between seed priming, phytochemical accumulation, and bioactivity, the underlying molecular and enzymatic mechanisms were not directly investigated. Future studies incorporating enzyme activity assays, transcriptomic analysis, and targeted metabolomics are therefore necessary to validate the mechanistic basis of the observed responses. Incorporating additional antioxidant assays such as 2,2′-azinobis-(3-ethylbenzothiazoline-6-sulfonic acid) radical scavenging assay, ferric reducing antioxidant power assay, and oxygen radical absorbance capacity assay is important to provide a more comprehensive evaluation of antioxidant capacity beyond DPPH. It is also recommended to test the extracts against a broader range of bacterial strains, including both Gram-positive and Gram.

## Data Availability

The original contributions presented in the study are included in the article/supplementary material. Further inquiries can be directed to the corresponding author.
